# The Oncogenic Effects, Pathways, and Target Molecules of JC Polyoma Virus T Antigen in Cancer Cells

**DOI:** 10.3389/fonc.2022.744886

**Published:** 2022-03-08

**Authors:** Hua-Chuan Zheng, Hang Xue, Yu-Zi Jin, Hua-Mao Jiang, Zheng-Guo Cui

**Affiliations:** ^1^Department of Oncology and Experimental Center, The Affiliated Hospital of Chengde Medical University, Chengde, China; ^2^Department of Pediatrics, The Affiliated Hospital of Chengde Medical University, Chengde, China; ^3^Department of Urology, The First Affiliated Hospital of Jinzhou Medical University, Jinzhou, China; ^4^Department of Environmental Health, University of Fukui School of Medical Science, Fukui, Japan

**Keywords:** JC virus T antigen, oncogenesis, signal pathway, WNT/beta-catenin pathway, PI3k-Akt signal pathway

## Abstract

JC polyoma virus (JCPyV) is a ubiquitous polyoma virus that infects the individual to cause progressive multifocal leukoencephalopathy and malignancies. Here, we found that T-antigen knockdown suppressed proliferation, glycolysis, mitochondrial respiration, migration, and invasion, and induced apoptosis and G_2_ arrest. The reverse was true for T-antigen overexpression, with overexpression of Akt, survivin, retinoblastoma protein, β-catenin, β-transducin repeat-containing protein (TRCP), and inhibitor of growth (ING)1, and the underexpression of mammalian target of rapamycin (mTOR), phosphorylated (p)-mTOR, p-p38, Cyclin D1, p21, vascular endothelial growth factor (VEGF), ING2, and ING4 in hepatocellular and pancreatic cancer cells and tissues. In lens tumor cells, T antigen transcriptionally targeted viral carcinogenesis, microRNAs in cancer, focal adhesion, p53, VEGF, phosphoinositide 3 kinase-Akt, and Forkhead box O signaling pathways, fructose and mannose metabolism, ribosome biosynthesis, and choline and pyrimidine metabolism. At a metabolomics level, it targeted protein digestion and absorption, aminoacryl-tRNA biosynthesis, biosynthesis of amino acids, and the AMPK signal pathway. At a proteomic level, it targeted ribosome biogenesis in eukaryotes, citrate cycle, carbon metabolism, protein digestion and absorption, aminoacryl-tRNA biosynthesis, extracellular-matrix-receptor interaction, and biosynthesis of amino acids. In lens tumor cells, T antigen might interact with various keratins, ribosomal proteins, apolipoproteins, G proteins, ubiquitin-related proteins, RPL19, β-catenin, β-TRCP, p53, and CCAAT-enhancer-binding proteins in lens tumor cells. T antigen induced a more aggressive phenotype in mouse and human cancer cells due to oncogene activation, inactivation of tumor suppressors, and disruption of metabolism, cell adhesion, and long noncoding RNA-microRNA-target axes.

## Introduction

The JC polyoma virus (JCPyV) belongs to the human DNA polyoma virus family together with BK and SV40 polyoma viruses. It is considered as an etiologic agent of progressive multifocal leukoencephalopathy (PML) and is a neurotropic virus. The replication of JCPyV is regulated by transcription factors (e.g., Jun, Sp1, nuclear factor-1, GF-1, Sμbp-2, Purα, and Y-box-binding protein-1), especially in glial cells and neurons (1, 2). Of its encoded products, T antigen is an oncogenic and multifunctional phosphoprotein responsible for viral DNA replication by unwinding the double helix and recruiting ATPase, helicase, and polymerase (3). The T-antigen origin-binding domain has a C-terminal pocket where both A1 and B2 motifs exist. Its F258 region is highly dynamic and responsible for DNA binding (4). Its N-terminal phosphorylation at threonine 125 is likely mediated by Cyclin-CDK, critical to binding retinoblastoma protein (Rb) p107 and p130, and maintaining Rb-E2F complexes (5). In glial cells, T antigen promotes viral gene expression by inhibiting SRSF1 transcription *via* interaction with the promoter region of SRSF1 (6). Interferon-γ inhibits JCPyV replication by suppressing the expression of T antigen (7). *In vivo*, p53 blocks JCPyV DNA replication *via* interaction with T antigen (8). In turn, T-antigen expression is suppressed by glucose deprivation in both medulloblastoma cells and glioblastoma xenografts, which partly results from 5′-activated AMP kinase, and the production of reactive oxygen species (9).

Reportedly, JCPyV might use extracellular vesicles to infect target cells independently of virus receptors (10). In addition, phosphoinositide 3 kinase (PI3K)γ facilitated the infection of human SVG-A glial cells by JCPyV (11). Mayberry et al. (12) found that JCPyV entry by clathrin-mediated endocytosis was driven by β-arrestin. G protein-coupled receptor kinase 2 was found to mediate β-arrestin interactions with 5-hydroxytryptamine receptors for the clathrin-mediated endocytosis of JCPyV (13, 14). After entry into host cells, JCPyV T antigen activated the antiapoptotic survivin promoter; T antigen bound to survivin and translocated it to the nucleus for the development of PML (15). Recently, the T antigen of JCPyV has been closely linked to the tumorigenesis of oral and urothelial carcinomas, as well as esophageal, gastric, colorectal, anal, head neck squamous, lung, prostatic, and breast cancers (1–3). Transgenic mice expressing JCPyV T antigen *via* a Mad-1 promoter developed malignant peripheral nerve sheath tumors and pituitary adenomas (1). Our group has established various transgenic mice overexpressing T antigen and observed the development of either lens or pulmonary tumor (16, 17). However, the oncogenic mechanisms of T antigen remain elusive. Herein, we aimed to clarify the target molecules and signaling pathways of T antigen using cell function assays, transgenic mice, and multiomics analysis.

## Materials and Methods

### Cell Culture and Plasmid Transfection

Primary culture of lens tumor cells from α-crystallin-JCPyV T-antigen transgenic mouse was carried out as previously described (16, 18). Mouse lens tumor, hepatocellular (QGY7703), and pancreatic carcinoma (PANC1) cells were grown in DMEM medium mixed with 10% fetal bovine serum (FBS), penicillin (100 units/ml), and streptomycin (100 μg/ml) in a humidified atmosphere of 5% CO_2_ at 37°C. Lens tumor cells were subjected to shRNA-T-antigen transfection, and QGY7703 and PANC cells to green fluorescent protein (GFP)-tagged JCPyV T antigen-expressing plasmid. shRNA-T antigen targets: sense, 5′-GCAGGUUUCAUGGAAAUUAdTdT-3′ and anti-sense: 5′-UAAUUUCCAUGAAACCUGCdTdT-3′ (799-821).

### Proliferation

We used cell counting kit-8 (CCK-8) to detect cellular viability. Briefly, we cultured 2.5 × 10^3^ cells/well on a 96-well plate until adherence. At different times, 10 μl of CCK-8 solution was dispensed into each well, incubated for 3 h, and read at 450 nm on a spectrophotometer.

### Metabolism Assay

We dispensed 4,000 cells/well into the plates of XF-24 Extracellular Flux Analyzers (Seahorse Bioscience, North Billerica, MA, USA) until adherence. Extracellular acidification and oxygen consumption rates were determined in XF media (DMEM containing 1 mM sodium pyruvate, either 10 or 15 mM glucose, and 2 mM l-glutamine) under basal conditions or in the presence of mitochondrial inhibitors (1 mM antimycin A + 1 mM oligomycin and/or 100 nM rotenone).

### Cell-Cycle Analysis

Cells (1 × 10^6^) were trypsinized, pelleted, washed by phosphate-buffered saline (PBS), and fixed in cold 75% ethanol. Cells were subsequently washed in PBS and treated with RNase A. Cells were then gently mixed with propidium iodide (PI, 50 µg/ml). Finally, flow cytometry was used to detect PI signal.

### Apoptosis Assay

We stained cells using PI and PE-labeled Annexin-V 7-AAD to detect phosphatidylserine externalization as a marker of early apoptosis according to the manufacturer’s recommended protocol (Keygen, Nanjing, China).

### Cell Migration and Invasion Assays

To assay migration, we dispensed 50,000 cells suspended in serum-free medium into a membrane insert (Corning, Corning, NY, USA) sitting on top of a transwell chamber with 10% FBS. After a 24-h incubation, cells on the membrane were scrubbed off and the membrane washed in PBS, fixed in methanol, and stained with crystal violet. Similar procedures were adopted for invasion assays, which included a matrigel coating in the insert.

### Coimmunoprecipitation

For coimmunoprecipitation (co-IP), more than 1 mg of a protein sample was precleared with 50 μl protein A-sepharose beads for 60 min with gentle rotation, and then incubated with 5 μg primary antibody (**Supplementary Table S1**) in a 1-ml mixture overnight on a rotator. Subsequently, 100 μl protein A sepharose beads (50% slurry) were added, and the samples were rotated at 4°C overnight. The sample was then centrifuged for 10 min at 4,000×*g* to remove nonspecific binding proteins. The beads were washed five times with 1% NP40 lysis buffer. The pellet was eluted by adding 50 μl 2 × sodium dodecyl sulfate (SDS) sample buffer and then heated at 100°C for 20 min. The samples were pelleted to remove the beads, and the supernatant was prepared for gel electrophoresis and Western blot.

### Animal Model

Three mice were maintained in each plastic cage covered with corn cob chips in specific pathogen-free conditions, temperature control, and a 12-h light/dark cycle. Mouse food intake consisted of standard rodent food and microbe-free water. Protocols for all mouse experiments were approved by the Animal Experimentation Committee of The Affiliated Hospital of Chengde Medical University. Transgenic mice with CAG-loxp-LacZ/JCPyV T antigen were generated at Shanghai Biomodel Organism Science & Technology Development Co. Ltd. To activate T-antigen expression in pancreatic ductal epithelia or hepatocytes, we crossed CAG- loxp-LacZ T antigen mice with Pdx1-cre (specific for pancreas, Jax Lab) and Alb-cre (specific for hepatocytes, Jax Lab) mice to establish spontaneous tumor models.

### Preparation of RNA-Seq Libraries, Sequencing

Total RNA samples were extracted, treated with DNase I, and purified by oligo-T magnetic beads. After that, mRNA was degraded into short fragments and converted into single-stranded and then double-stranded cDNA. We prepared ends and added 3′-end single adenine for the ligation of sequencing adaptors, followed by PCR amplification. Library amplicons were sequenced by an Illumina HiSeq™ 4000 (San Diego, CA, USA), and raw data were aligned to known sequences.

### Mirna Sequencing

Small RNAs (18–30 bases) were enriched by polyacrylamide gel electrophoresis (PAGE) and ligated with 3′-adapters. RNAs (36–44 bases) were enriched, and then ligated to 5′-adapters. The ligation samples were amplified by reverse transcription (RT)-PCR, and amplicons with 140–160 bp were enriched, and sequenced using an Illumina HiSeq™ 4000. Raw data were analyzed using Illumina Genome Analyzer Pipeline software.

### LN-RNA Sequencing

Total RNA was subjected to the removal of ribosomal RNA to retain mRNAs and noncoding (nc)RNAs, which were degraded into short fragments and converted into single-stranded and then double-stranded cDNA. The final cDNA samples were purified, end-repaired, poly (A) added, and ligated to Illumina sequencing adapters. Uracil-N-glycosylase was used to digest the double-stranded cDNA and these fragments were purified by agarose gel electrophoresis, amplified by PCR, and sequenced by an Illumina HiSeq™ 4000.

### Metabolomics Analysis

Each sample was homogenized, frozen in order to precipitate proteins, and centrifuged. The supernatant was subjected to vacuum concentration and mixed by extraction liquid reconstitution. After centrifugation, the supernatant was analyzed by ultra-high-performance liquid chromatography quadrupole time-of-flight mass spectrometry. The preprocessing results consisted of the retention time, peak intensity, and mass-to-charge ratio (m/z) values. R package CAMERA was employed for peak annotation after XCMS data processing. An in-house MS2 database was applied to identify metabolites. The data were dealt with using partial least squares and orthogonal projection to latent structures-discriminant analyses.

### Proteomic Analysis by iTRAQ Labeling and Two-Dimensional Nano-LC-MS/MS

For isobaric tags for relative and absolute quantification (iTRAQ) analysis, protein samples were extracted from lens tumor cells and sh-T transfectants in radioimmunoprecipitation assay lysis buffer containing cocktail protease inhibitors (Roche, Basel, Switzerland). Each sample (200 μg) was digested and labeled with the following iTRAQ labels. Samples were separated by strong cation exchange and sequentially analyzed by two-dimensional liquid chromatography and tandem mass spectrometry (LC-MS/MS). Data were acquired under an information-dependent acquisition mode, with a dynamic exclusion set to exclude any m/z values that had been picked for the MS/MS scan.

### Western Blot

Protein samples were extracted in radio-immunoprecipitation assay lysis buffer, subjected to concentration, separated by SDS-PAGE, and transferred to a Hybond membrane, which was then blocked in 5% skim milk in tris-buffered saline and Tween 20 (TBST) for 1 h. For immunoblots, the membrane was incubated for 1 h with primary antibody (**Supplementary Table S1**) and then with IgG conjugated to horseradish peroxidase (DAKO, Glostrup, Denmark) in TBST. We observed bands using ECL detection reagents.

### Statistical Analysis

For bioinformatics analysis, we performed downstream analysis according to gene expression (principal component analysis/correlation/screening differentially expressed genes) and Gene Ontology and Kyoto Encyclopedia of Genes and Genomes (KEGG) pathway enrichment analyses. We also carried out Mann–Whitney *U* tests to compare means. Two-sided *p <* 0.05 was regarded as statistically significant. SPSS 10.0 software was used for the aforementioned statistics.

## Results

### The Roles of T-Antigen Expression in Tumor Cells

**Figure 1:**
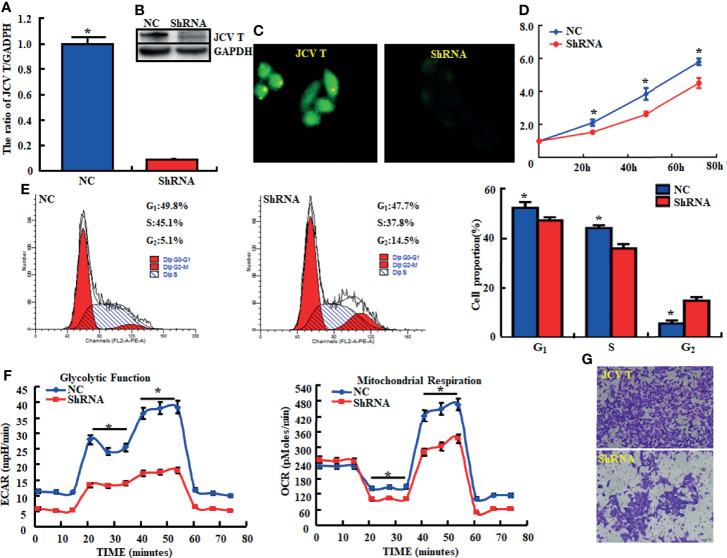
The effects of T antigen knockdown on the aggressive phenotypes of lens tumor cells. After transfection of short-hairpin (sh)RNA-T antigen, its expression became weak in lens tumor cells as determined by real-time reverse transcription (RT)-PCR **(A)**, Western blot **(B)**, and immunofluorescence **(C)**. Cell viability in lens tumor cells and their transfectants were measured using a cell counting kit-8 (CCK-8) kit **(D)**. Cell cycle, apoptosis, glucose metabolism, and invasion were examined by propidium iodide (PI, **E**), XF-24 extracellular flux analyzers **(F)**, and transwell assays **(G)**, respectively. ^*^p < 0.05, compared with transfectants. NC, negative control.

**Figure 2:**
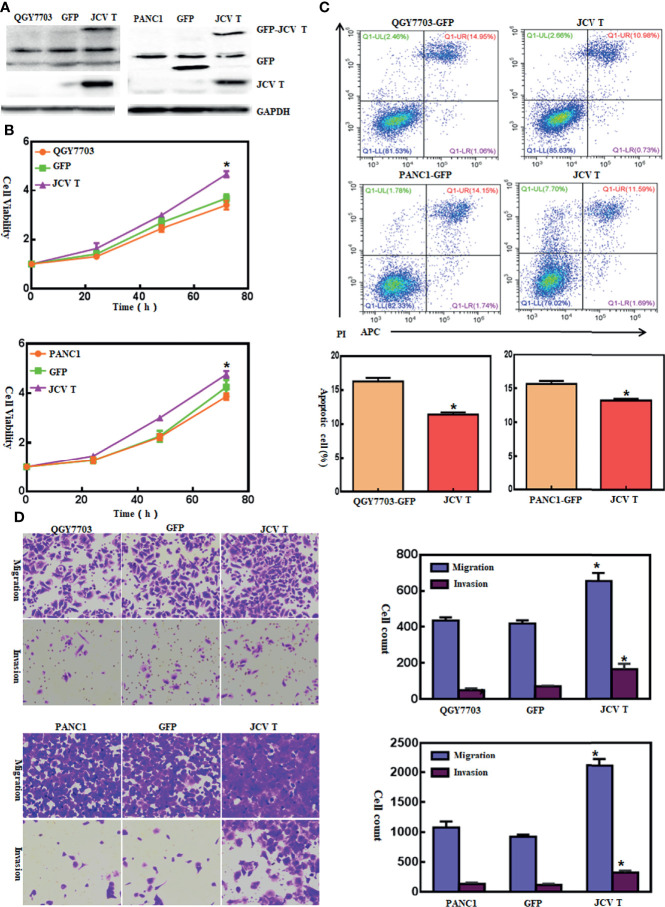
The effects of T-antigen overexpression on the aggressive phenotypes of cancer cells. After transfection of pEGFP-N1-T antigen, strong expression was observed in QGY7703 and PANC cells as determined by Western blot **(A)**. Cell viability in both cancer cells and their T-antigen transfectants was measured using a cell counting kit-8 (CCK-8) kit **(B)**. Apoptosis, migration, and invasion were measured by Annexin-V staining **(C)** and transwell chamber **(D)** assays, respectively. ^*^p < 0.05, compared with control and mock.

Here, we successfully cultured lens tumor cells and then knocked down T antigen using short hairpin RNA as revealed by Western blot and immunofluorescence staining (**Figure 1A**). T-antigen underexpression reduced proliferation, glycolysis, mitochondrial respiration, and invasion and promoted apoptosis and G_2_ arrest (**Figures 1B–G**, *p* < 0.05). In QGY7703 and PANC1 cancer cells, T-antigen overexpression increased cellular proliferation, decreased apoptosis, and promoted migration and invasion (**Figure 2**, *p* < 0.05).

### The Effects of T Antigen on Transcriptome of Lens Tumor Cells

**Figure 3:**
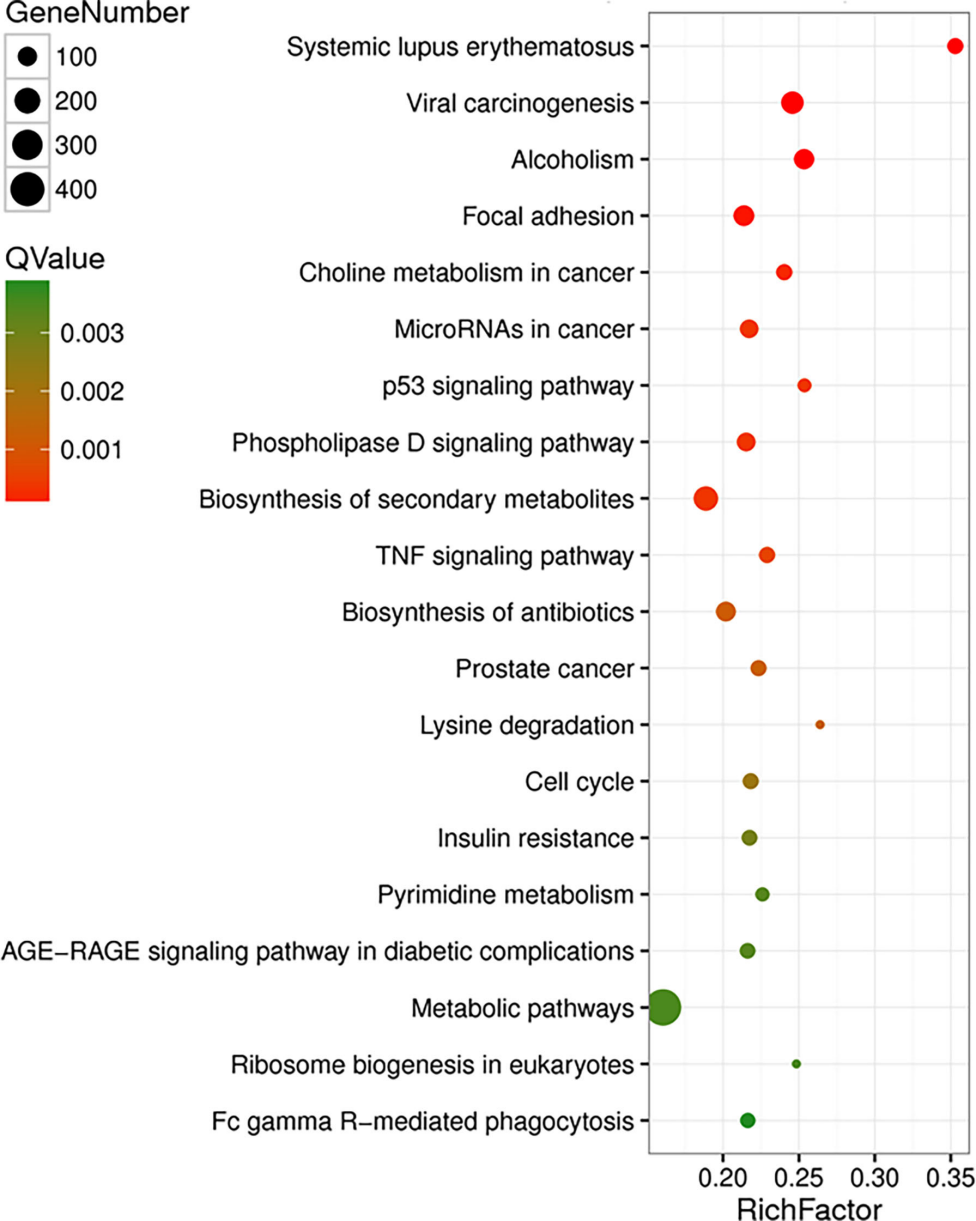
The effects of T antigen on transcriptome of lens tumor cells. The top signaling pathways for upregulated and downregulated genes after T-antigen knockdown in lens tumor cells are listed according to the Kyoto Encyclopedia of Genes and Genomes (KEGG) analysis.

In lens tumor cells, upregulated and downregulated genes after T-antigen knockdown are outlined in **Supplementary Tables S2, S3**, respectively. They were classified into molecular function, cellular component, and biological process according to Gene Ontology analysis. The top signaling pathways, according to KEGG analysis, are shown in **Figure 3**. The important pathways were viral carcinogenesis, micro(mi)RNAs in cancer, focal adhesion, p53 signaling pathways, cell cycle, ribosome biosynthesis, insulin resistance, tumor necrosis factor signaling pathway, phosphoinositide 3 kinase (PI3K)-Akt signaling pathway, vascular endothelial growth factor (VEGF) signaling pathway, fructose and mannose metabolism, biosynthesis of amino acids, RNA transport, adherens junction, proteoglycans in cancer, aminoacryl-tRNA biosynthesis, choline metabolism in cancer, pyrimidine metabolism, and Forkhead box O (FoxO) signaling pathway (**Supplementary Table S4**). The top upregulated and downregulated miRNAs and long noncoding (lnc)RNAs are listed in **Supplementary Tables S5–S8**, respectively.

### The Effects of T Antigen on Metabolome of Lens Tumor Cells

**Figure 4:**
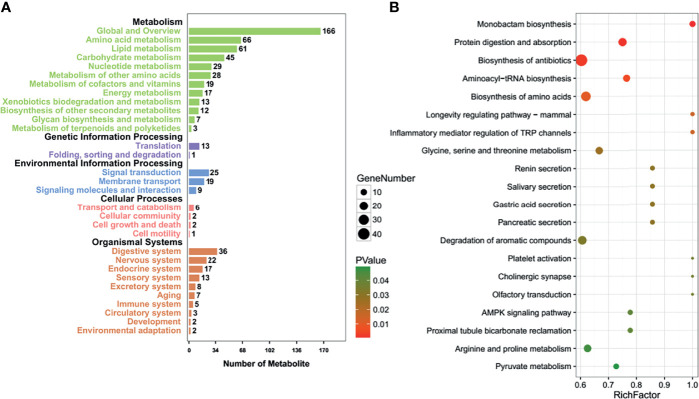
The effects of T antigen on metabolome of lens tumor cells. Relevant Kyoto Encyclopedia of Genes and Genomes (KEGG) pathways after T-antigen knockdown in lens tumor cells were classified into metabolism, genetic information processing, environmental information processing, cell processes, or organismal systems **(A)**. The top signaling pathways are listed according to the KEGG analysis **(B)**.

The pathways involved after T-antigen silencing in lens tumor cells were classified into metabolism (amino acid, lipid, nucleotide, vitamin, energy, xenobiotics, glycan and terpenoid, and polyketides), genetic information processing (translation, folding, sorting, and degradation), environmental information processing (membrane transport, signaling molecules, and interaction), cell processes (transport and catabolism, cellular community, growth, death, and motility), and organismal systems (nervous system, sensory system, environmental adaptation, and the like; **Figure 4A**). The top signaling pathways are shown in **Figure 4B**. The important pathways, according to KEGG analysis, included the longevity regulating pathway, protein digestion and absorption, aminoacryl-tRNA biosynthesis, biosynthesis of amino acids, cholinergic synapse, and AMP-activated protein kinase (AMPK) signaling pathway (**Supplementary Table S9**).

### The Effects of T Antigen on Proteome in Lens Tumor Cells and Spontaneous Tumors

Isobaric tags for relative and absolute quantitation-labeling LC-MS/MS analyses identified top differential proteins in T-antigen-silenced transfectants as shown in **Supplementary Table S10** (upregulated) and **Supplementary Table S11** (downregulated). Cluster of orthologous groups of protein functions mainly consisted of RNA processing and modification, nuclear and chromatin structure, energy production and conversion, cell cycle and division, the metabolism of amino acid, nucleotide, carbohydrate, lipid, secondary metabolites and coenzyme, gene expression process (replication-transcription-translation-posttranslational modification), and extracellular matrix and cell mobility. Biological processes consisted of growth, adhesion, cellular component organization or biogenesis, and metabolism, cell components did extracellular matrix and region, membrane, transmembrane complex, and lumen, and molecular function did binding, catalyzation, and transcriptional factor and transporter. The top canonical pathway analyses indicated significant differences in ribosome biogenesis in eukaryotes, citrate cycle, carbon metabolism, protein digestion and absorption, extracellular-matrix (ECM)-receptor interaction, aminoacryl-tRNA biosynthesis, and biosynthesis of amino acids (**Supplementary Table S12**).

**Figure 5:**
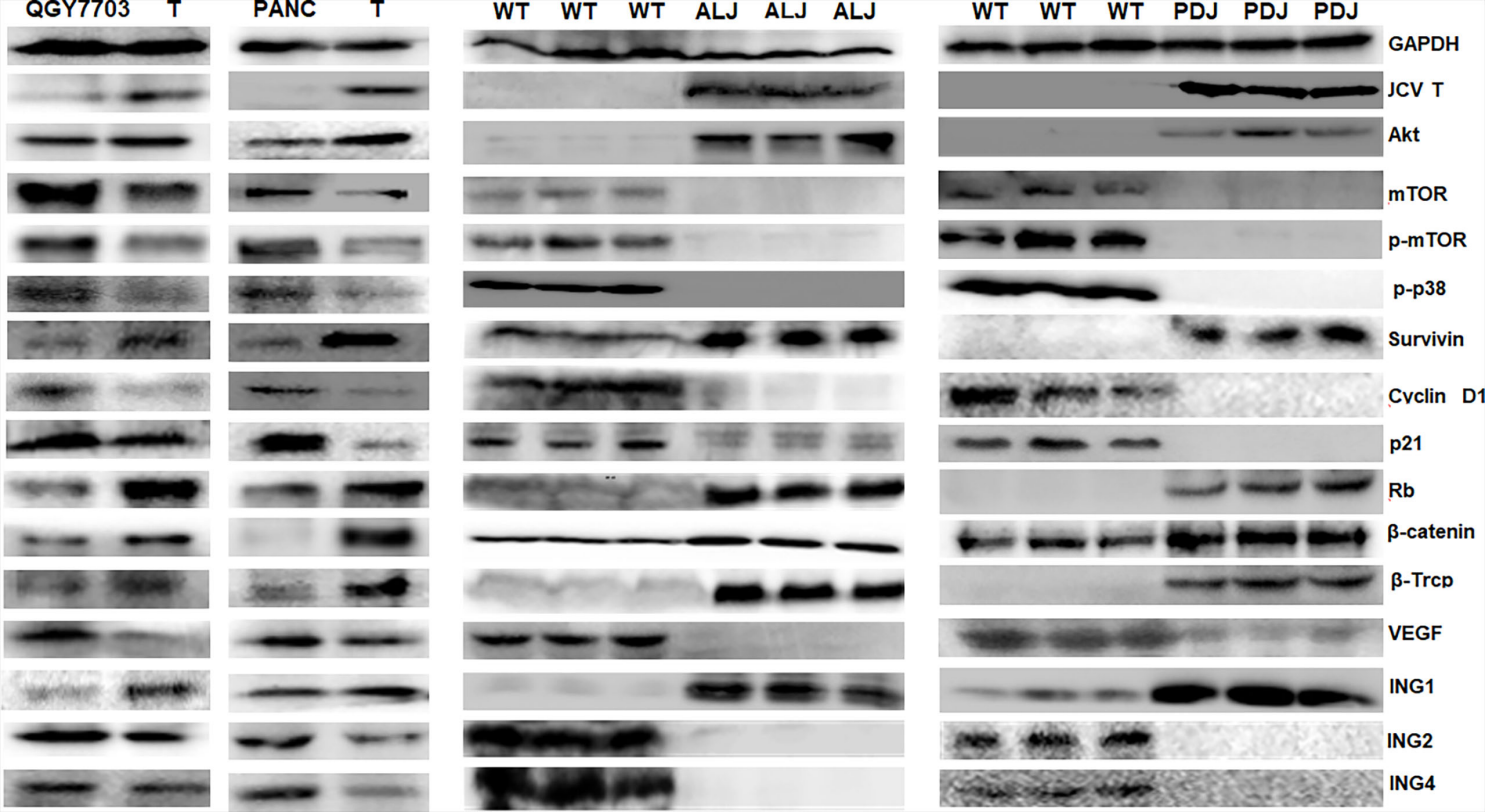
The effects of T antigen on protein expression in cancer cells and spontaneous tumors. Western blot was employed to examine the distinct protein expression in T-overexpressing QGY-7703 and PANC-1 cells, and in spontaneous hepatocellular carcinoma or pancreatic adenocarcinomas of Alb-cre/T antigen (ALJ) and Pdx1-cre/T antigen (PDJ) transgenic mice. Parental cells and liver and pancreas of wild-type (WT) mice were used as controls.

Compared with the control, overexpression was observed of JCPyV T antigen, Akt, survivin, retinoblastoma protein (Rb), β-catenin, β-transducin repeat-containing protein (TRCP), and inhibitor of growth (ING) 1 in T-antigen-overexpressing hepatocellular and pancreatic cancer cells and spontaneous hepatocellular carcinoma or pancreatic adenocarcinoma tissues, while the underexpression of mammalian target of rapamycin (mTOR), p-mTOR, p-p38, Cyclin D1, p21, VEGF, ING2, and ING4 was noted (**Figure 5**).

### The Partner Proteins of T Antigen in Lens Tumor Cells

**Figure 6:**
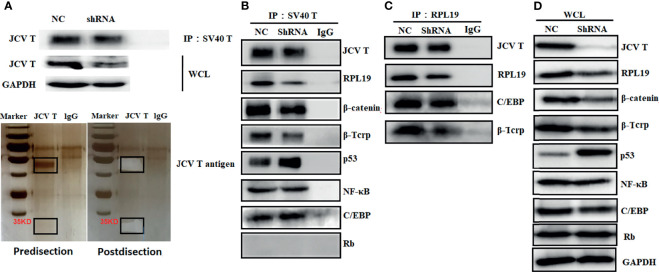
The partner proteins of JCV T antigen in lens tumor cells. Proteins from T antigen-silenced lens tumor cells were subjected to coimmunoprecipitation (co-IP) by anti-SV40 T-antigen antibody. Co-IP samples were analyzed by Western blot and by electrophoresis and silver staining **(A)**. Protein samples from anti-SV40 T-antigen antibody co-IP were screened for binding proteins to T antigen **(B)**. Protein samples from co-IP using anti-RPL19 antibody were screened for interacting proteins **(C)**. Western blot was used to detect whole cell lysates as an internal control **(D)**. NC, negative control; WCL, whole cell lysates.

Protein samples from control and T-antigen-silenced lens tumor cells were subjected to co-IP using anti-SV40 antibody, and co-IP samples underwent gel electrophoresis and silver staining (**Figure 6A**). A 35-kD band was dissected from the gel and analyzed by protein flight mass spectroscopy. As shown in **Supplementary Table S13**, the band contained various keratins, ribosomal proteins, apolipoproteins, G proteins, and ubiquitin-related proteins, as well as other proteins. To verify these proteins, we carried out a co-IP that suggested T antigen might interact with ribosomal protein (RPL) 19, β-catenin, β-TRCP, p53, and CCAAT-enhancer-binding proteins (C/EBP) in lens tumor cells; the interactions were consistent with protein expression levels after T-antigen silencing. Moreover, RPL19 bound to T antigen, C/EBP, and β-TRCP (**Figures 6B–D**).

## Discussion

Link et al. (19) found that T-antigen-expressing colorectal cancer cells showed a two- to threefold increase in migration and invasion, with Akt- and mitogen-activated protein kinase (MAPK) signaling pathways activated. Huang et al. (20) demonstrated that small T-antigen-overexpressing lung cancer cells showed lower nucleotide excision repair activity, more DNA damage foci, and increased sensitivity to ultraviolet light and cisplatin, as well as chromosomal breakages. In the present study, we used a primary culture of lens tumor cells as a T-antigen-positive cell model and subsequently silenced T-antigen expression, which ameliorated cellular proliferation, glycolysis, mitochondrial respiration, migration, and invasion. Additionally, we overexpressed T antigen in hepatocellular and pancreatic cancer cells, which promoted proliferation, migration, and invasion. Taken together, this suggests that T antigen can insert itself into genomic DNA to express an oncoprotein, which is responsible for the aggressiveness of cellular phenotypes

In the nucleus, T antigen can inactivate p53 and pRb proteins, while in the cytoplasm, it disrupts insulin-like growth factor I receptor (IGF-IR) signaling pathways to deregulate the cell cycle and subsequently proliferation (1–3). With regard to T-antigen-related signaling pathways, multiomics analysis showed that viral carcinogenesis included disrupted AMPK, Forkhead box O, and PI3K-Akt and p53 signaling pathways. In addition, T antigen was found to cause the overexpression of Akt, survivin, and Rb and the underexpression of p21. These findings indicate that T antigen might activate Akt/NF-κB/survivin leading to an antiapoptosis effect and Rb overexpression and p21 underexpression for cell-cycle progression.

The ING family of tumor suppressors includes ING1–5, with the first identification of ING3 guiding the discovery of ING1–2 and 4–5. Their nuclear localization sequence favors nucleic translocation; the leucine zipper-like motif mediates interaction with leucine zipper proteins, and the plant homeodomain motif can bind to chromatin-interacting proteins such as methylated H3 histone. Indeed, both ING1 and ING2 have been involved in mSin3A/histone deacetylase complex formation. While ING3 complexes with hNuA4 histone acetyltransferase (HAT), both ING4 and ING5 bind with HBO1 HAT, but only ING5 binds with a MOZ/MORF HAT complex (21). Here, we, for the first time, found that T antigen upregulated ING1 expression but downregulated the expression of ING2 and ING4. These paradoxical results might be explained by the effects of T antigen on transcription *via* interaction with their promoter and on their proteasomal degradation since JCPyV T antigen was shown to interact with ubiquitin ligase E3 β-TRCP1 and phosphodegron (DpSGX(2-4)pS) to suppress proteasomal degradation (22).

The physical interaction between the central domain of T antigen and the C-terminus of β-catenin elevates the amount of β-catenin due to the high stability of the protein and facilitates its nuclear import (23). Bhattacharyya et al. (24) have identified that T antigen recruited Rac1 to stabilize β-catenin by suppressing its ubiquitin-dependent proteasomal degradation. The nuclear colocalization of both T antigen and β-catenin significantly enhanced T-cell factor-dependent promoter activity and activated c-Myc and Cyclin D1 (25). T antigen bound to β-TRCP and influenced the stability of β-catenin (22). Here, we observed that T antigen interacted with β-catenin and β-TRCP and strengthened their expression, suggesting positive effects of T antigen on the Wnt/β-catenin pathway. In the present study, T antigen might downregulate the expression of Cyclin D1, accounting for the underexpression of downstream VEGF in T antigen-overexpressing cells.

A ribosome serves as an organelle for protein synthesis and is composed of 40S small and 60S large subunits, including a variety of ribosomal proteins (26). The 60S ribosomal protein L19 (RPL19) belongs to the ribosomal protein L19 family, contains 196 amino acids, and functions as a component of the 60S subunit (26). RPL19 protein expression was regarded as a prognostic marker for prostate cancer (27), and fecal RPL19 mRNA was found to be a biomarker for aggressive behavior and is associated with an unfavorable prognosis in colorectal cancer (28). RPL19 knockdown influenced neither proliferation nor apoptosis, but reduced tumor growth *via* the regulation of cellular adhesion (29). Hong et al. (30) found that RPL19 strengthened the unfolded protein response and made MCF7 breast cancer cells sensitive to ER stress-induced cell death. Here, we demonstrated that the top pathway was ribosome biogenesis in eukaryotes in T-antigen-silenced lens tumor cells. Moreover, we observed that T antigen bound to RPL13, RPL15, RPL19, RPL29, and RPL25. Of these, RPL19 was verified by co-IP using RPL19 and T-antigen antibodies, suggesting the promoting effects of T antigen on ribosome biosynthesis.

In the present study, focal adhesion, cell mobility, and ECM-receptor interaction were found to be closely linked to JCPyV T antigen in lens tumor cells. Importantly, partner proteins of T antigen included keratin, type I cytoskeletal 10, 14, and 15, keratin, type II cytoskeletal 1, 2, 5, and 79, keratin Kb40, rho-related GTP-binding protein RhoC precursor, ras-related protein Rab-11A, Rab-14, and rho GDP-dissociation inhibitor 1, which increased cellular migration and invasion as mentioned above. Previously, our data showed that lung cancer with higher JCPyV copy numbers displayed a downregulation of cell adhesion mediated by membrane β-catenin (31). JCPyV T antigen expression promoted the invasion and migration of colorectal cancer cells *via* an Akt/MAPK signaling pathway and was found to be higher in metastatic primary and secondary tumors (19). In summary, T antigen can enhance the ability of cancer cells to migrate and invade *via* complex formation with both keratins and rho proteins. No further study about the effects of T antigen and their partner proteins on the migration and invasion is a limitation.

Cancer cells reprogram glycolysis to promote aggressive carcinogenesis by remodeling carbon flux into the serine biosynthetic pathway (32). In line with the promoting effects of T antigen on glucose metabolism of lens tumor cells, T antigen prevented reactive oxygen species release, a cytotoxicity induced by glucose deprivation, and promoted ATP production. Furthermore, the pentose phosphate inhibitors, 6-aminonicotinamide and oxythiamine, and the glycolytic inhibitor, 2-deoxy-d-glucose (AMPK activator), ameliorated the expression of T antigen, which regulated the expression of glycolytic hexokinase 2 and transaldolase-1 for the pentose phosphate pathway (19). Here, we found that T antigen was involved in glycolysis, mitochondrial respiration, and amino acid, pyrimidine, and choline metabolism. Previously, we demonstrated that SV40 polyoma virus T antigen might disrupt metabolism involving carbohydrates, amino acids, and nucleotides in the spontaneous lens tumor of transgenic mice (33) in line with the present findings. The two targets of mTOR (p70S6 kinase and 4EP1/2) modulate ribosome biogenesis and the metabolism of glucose and lipids (34). In this study, low expression of mTOR and its phosphorylated form in T-expressing spontaneous liver and pancreatic tumors might account for the dysfunctional effects of T antigen on biosynthesis, which was seen in our investigation.

The JCPyV miRNAs, JC-miR-3p and JC-miR-5p, were expressed early postinfection (35). Takahashi et al. (36) detected JCPyV-encoded miR-J1 in the nuclei of JCPyV-positive cells in PML. The measurement of miR-5p in urine might be useful as a biomarker to monitor JCPyV infection and to better identify the possible risk of developing PML in natalizumab-treated patients with multiple sclerosis (37). JC-miR-5p in saliva was used to evaluate JCPyV infection after renal transplantation (38) Link et al. (39) found miR-5p in either tumor or fecal samples was a potential biomarker for JCPyV infection in colorectal cancer. With regard to its transcription, miRNA in cancer was found in a T-antigen-knockdown lens tumor. In combination with the miRNA data in our study, an LncTar web was employed to predict the competition between lncRNAs and miRNAs. Targetscan and miRDB were used to predict the interaction between miRNAs and target proteins in the significant differential expression of lncRNAs, miRNAs, and proteins after T-antigen knockdown. Here, we found that Snhg17/hsa-miR-342-5p/NAA10, Malat1/hsa-miR-96-5p/SNAI2, miR-96-5p/Oxsr1, miR-96-3p/Tmem33, mmu-miR-190a-3p/Epb41l1 or Arhgap6, mmu-miR-190b-3p/Eml2, mmu-miR-6916-3p/Tmem33, mmu-miR-6916-5p/Kirrel, miR-1192/Lgmn or Smc5, mmu-miR-9-3p/Rfx5, mmu-miR-9b-3p/Mef2a, and mmu-miR-203-5p/Cdk8 were involved in the oncogenic effects of T antigen. However, further investigations are required using transgenic spontaneous tumors and their primary cells.

In summary, JCPyV T antigen aggravated aggressive phenotypes regardless of whether these were spontaneous mouse tumor or human cancer cells. JCPyV T antigen was closely linked to the activation of oncogene proteins and inactivation of tumor suppressors and disrupted metabolism, cell adhesion, and lncRNA-miRNA-target axes *via* transcriptional or translational regulation, protein degradation, and protein interactions. In the future, we should investigate the molecular and pathological features and individual therapies of JCPyV-related cancers. It is helpful to block JCPyV infection for children and even adults.

## Data Availability Statement

The original contributions presented in the study are publicly available. This data can be found here: https://db.cngb.org/cnsa/ accession number CNP0002714.

## Ethics Statement

The animal study was reviewed and approved by The Affiliated Hospital of Chengde Medical University.

## Author Contributions

H-CZ designed the study. HX and Y-ZJ carried out the experiment. H-MJ and Z-GC revised the draft. All authors listed have made a substantial, direct, and intellectual contribution to the work and approved it for publication.

## Funding

The present study was supported by an Award for Liaoning Distinguished Professor, Natural Science Foundation of Hebei Province (202130821010436) and National Natural Scientific Foundation of China (81672700).

## Conflict of Interest

The authors declare that the research was conducted in the absence of any commercial or financial relationships that could be construed as a potential conflict of interest.

## Publisher’s Note

All claims expressed in this article are solely those of the authors and do not necessarily represent those of their affiliated organizations, or those of the publisher, the editors and the reviewers. Any product that may be evaluated in this article, or claim that may be made by its manufacturer, is not guaranteed or endorsed by the publisher.
