# Supplementary material for: The Oncogenic Effects, Pathways, and Target Molecules of JC Polyoma Virus T Antigen in Cancer Cells
Source: Front Oncol. 2022 Mar 8;12:744886. doi: 10.3389/fonc.2022.744886 (PMC8958009; doi:10.3389/fonc.2022.744886)
Supplement: Supplementary file 1 [file Table_1.docx]

**Table 1.** The antibodies used for immunofluorescence, western blot and immunoprecipitation

| **Num** | **Antibody** | **Species** | **Dilution** | **Company** | **Code number** |
| --- | --- | --- | --- | --- | --- |
| 1 | GAPDH（6C5） | rabbit | 1:2000 | santa cruz | sc-32233 |
| 2 | SV40 T(Pab 101) | mouse | 1:1000 | santa cruz | sc-147 |
| 3 | Akt(pan)(11E7) | rabbit | 1:1000 | CST | 4685 |
| 4 | mTOR (7C10) | rabbit | 1:1000 | CST | 2983 |
| 5 | Phospho-mTOR (Ser2448) (D9C2) | rabbit | 1:1000 | CST | 5536 |
| 6 | p38 MAPK (D13E1) | rabbit | 1:1000 | CST | 8690 |
| 7 | Phospho-p38 MAPK (Thr180/Tyr182) (D3F9) | rabbit | 1:1000 | CST | 4511 |
| 8 | Survivin (8E2) | mouse | 1:500 | santa cruz | sc-47750 |
| 9 | Cyclin D1(H-295) | rabbit | 1:700 | santa cruz | sc-753 |
| 10 | p21 (F-5) | mouse | 1:500 | santa cruz | sc-6246 |
| 11 | Rb (C-2) | mouse | 1:300 | santa cruz | sc-74562 |
| 12 | β-catenin (D10A8) | rabbit | 1:500 | CST | 8480 |
| 13 | β-Trcp (C-6) | mouse | 1:500 | santa cruz | sc-390629 |
| 14 | VEGF (C-1) | mouse | 1:500 | santa cruz | sc-7269 |
| 15 | ING1 | rabbit | 1:1000 | proteintech | 16185-1-AP |
| 16 | ING2 | rabbit | 1:1000 | proteintech | 11560-1-AP |
| 17 | ING4 | rabbit | 1:1000 | proteintech | 10617-1-AP |
| 18 | RPL19 | rabbit | 1:1000 | proteintech | 14701-1-AP |
| 19 | C/EBPα(D-5) | mouse | 1:300 | santa cruz | sc-365318 |
| 20 | p53 | rabbit | 1:1000 | proteintech | 10442-1-AP |
| 21 | NF-kBp65(D14E12) | rabbit | 1:1000 | CST | 8242 |

**Table 2.** The up-regulated top mRNAs after T antigen knockdown in lens tumor cells

| **Num** | **Symbol** | **Gene** | **JCVT_fpkm** | **ShJCVT_fpkm** | **log2(FC)** | **P value** |
| --- | --- | --- | --- | --- | --- | --- |
| 1 | Hist1h2br | H2B clustered histone 24 | 0.001 | 60.81 | 15.89202097 | 2.10E-283 |
| 2 | Ak6 | adenylate kinase 6 | 0.001 | 32.71 | 14.99744414 | 7.55E-152 |
| 3 | 9530068E07Rik | RIKEN cDNA 9530068E07 gene | 0.001 | 29.05 | 14.82625054 | 6.46E-267 |
| 4 | Actr3 | actin related protein 3 | 0.001 | 27.56 | 14.75028827 | 8.35E-261 |
| 5 | Serpine1 | serpin family E member 1 | 0.001 | 17.49 | 14.09424267 | 5.25E-178 |
| 6 | Matr3 | matrin 3 | 0.001 | 13.8 | 13.75238065 | 3.77E-98 |
| 7 | Hist1h2ah | histone cluster 1 H2A family member H | 0.001 | 13.7 | 13.74188827 | 3.68E-22 |
| 8 | Msantd3 | Myb/SANT DNA binding domain containing 3 | 0.001 | 13.38 | 13.7077905 | 7.37E-83 |
| 9 | Lgmn | legumain | 0.001 | 13.26 | 13.69479315 | 9.88E-94 |
| 10 | Rbmx | RNA binding motif protein X-linked | 0.001 | 13.11 | 13.67838007 | 1.77E-105 |
| 11 | Fam3c | FAM3 metabolism regulating signaling molecule C | 0.001 | 13.02 | 13.66844183 | 3.06E-81 |
| 12 | Sptan1 | spectrin alpha, non-erythrocytic 1 | 0.001 | 12.97 | 13.66289086 | 0 |
| 13 | Osbpl3 | oxysterol binding protein like 3 | 0.001 | 12.4 | 13.5980525 | 2.91E-137 |
| 14 | Ddx5 | DEAD-box helicase 5 | 0.001 | 11.64 | 13.50680344 | 6.98E-38 |
| 15 | Iars | isoleucine-tRNA synthetase | 0.001 | 11.55 | 13.49560523 | 6.12E-192 |
| 16 | Brox | BRO1 domain and CAAX motif containing | 0.001 | 11.51 | 13.49060021 | 1.10E-148 |
| 17 | Gm45837 |  | 0.001 | 10.59 | 13.37041497 | 1.15E-165 |
| 18 | Bace1 | beta-secretase 1 | 0.001 | 10.2 | 13.31628153 | 9.65E-156 |
| 19 | Dctd | dCMP deaminase | 0.001 | 9.75 | 13.2511865 | 1.51E-78 |
| 20 | Usp19 | ubiquitin specific peptidase 19 | 0.001 | 9.68 | 13.24079133 | 3.84E-165 |
| 21 | Zfp961 | zinc finger protein 961 | 0.001 | 9.56 | 13.2227949 | 2.84E-98 |
| 22 | Samd12 | sterile alpha motif domain containing 12 | 0.001 | 9.52 | 13.21674586 | 1.99E-38 |
| 23 | Zc3h11a | zinc finger CCCH-type containing 11A | 0.001 | 9.39 | 13.19690944 | 5.27E-156 |
| 24 | Vdac2 | voltage dependent anion channel 2 | 0.001 | 9.25 | 13.17523765 | 5.45E-35 |
| 25 | Srd5a3 | steroid 5 alpha-reductase 3 | 0.001 | 9.06 | 13.14529533 | 1.37E-44 |
| 26 | Cdk8 | cyclin dependent kinase 8 | 0.001 | 8.8 | 13.10328781 | 2.04E-100 |
| 27 | Ate1 | arginyltransferase 1 | 0.001 | 8.78 | 13.10000522 | 4.06E-52 |
| 28 | Eif4g1 | eukaryotic translation initiation factor 4 gamma 1 | 0.001 | 8.42 | 13.03960452 | 7.01E-100 |
| 29 | Mrpl52 | mitochondrial ribosomal protein L52 | 0.001 | 8.2 | 13.00140819 | 7.95E-14 |
| 30 | Fbxw2 | F-box and WD repeat domain containing 2 | 0.001 | 8.08 | 12.98013958 | 5.21E-60 |
| 31 | Mcam | melanoma cell adhesion molecule | 0.001 | 8.03 | 12.97118427 | 2.18E-92 |
| 32 | Mthfd1 | methylenetetrahydrofolate dehydrogenase, cyclohydrolase and formyltetrahydrofolate synthetase 1 | 0.001 | 7.97 | 12.96036401 | 1.57E-23 |
| 33 | Samd4 | sterile alpha motif domain containing 4 | 0.001 | 7.79 | 12.92740761 | 5.93E-201 |
| 34 | Crls1 | cardiolipin synthase 1 | 0.001 | 7.79 | 12.92740761 | 1.96E-22 |
| 35 | Snapc4 | small nuclear RNA activating complex polypeptide 4 | 0.001 | 7.73 | 12.9162527 | 5.61E-129 |
| 36 | Tank | TRAF family member associated NFKB activator | 0.001 | 7.66 | 12.90312868 | 1.50E-60 |
| 37 | Erlin1 | ER lipid raft associated 1 | 0.001 | 7.62 | 12.89557528 | 3.41E-93 |
| 38 | Cab39l | calcium binding protein 39 like | 0.001 | 7.43 | 12.8591465 | 6.86E-41 |
| 39 | Shoc2 | SHOC2 leucine rich repeat scaffold protein | 0.001 | 7.38 | 12.8494051 | 1.58E-114 |
| 40 | Hax1 | HCLS1 associated protein X-1 | 0.001 | 7.12 | 12.79766153 | 1.25E-24 |
| 41 | Gm14434 | predicted gene 14434 | 0.001 | 6.97 | 12.76694294 | 2.42E-40 |
| 42 | Smc5 | structural maintenance of chromosomes 5 | 0.001 | 6.97 | 12.76694294 | 2.18E-92 |
| 43 | Hist2h3c1 | H3 clustered histone 14 | 0.001 | 6.86 | 12.74399286 | 1.05E-48 |
| 44 | Wdr48 | WD repeat domain 48 | 0.001 | 6.69 | 12.7077905 | 3.13E-18 |
| 45 | Ube2e2 | ubiquitin conjugating enzyme E2 E2 | 0.001 | 6.63 | 12.69479315 | 1.37E-17 |
| 46 | Oxsr1 | oxidative stress responsive kinase 1 | 0.001 | 6.6 | 12.68825031 | 9.93E-118 |
| 47 | Tnnt2 | troponin T2, cardiac type | 0.001 | 6.59 | 12.68606275 | 3.43E-29 |
| 48 | Tipin | TIMELESS interacting protein | 0.001 | 6.49 | 12.66400276 | 4.26E-10 |
| 49 | Ube2j2 | ubiquitin conjugating enzyme E2 J2 | 0.001 | 6.29 | 12.6188443 | 1.92E-34 |
| 50 | Shisa5 | shisa family member 5 | 0.001 | 6.23 | 12.60501645 | 4.26E-10 |
| 51 | Cd2bp2 | CD2 cytoplasmic tail binding protein 2 | 0.001 | 6.2 | 12.5980525 | 4.87E-70 |
| 52 | Sspn | sarcospan | 0.001 | 6.12 | 12.57931594 | 1.75E-103 |
| 53 | Cd151 | CD151 molecule (Raph blood group) | 0.001 | 6.06 | 12.56510208 | 1.06E-38 |
| 54 | Nup188 | nucleoporin 188 | 0.001 | 6 | 12.55074679 | 1.27E-13 |
| 55 | Pcnp | PEST proteolytic signal containing nuclear protein | 0.001 | 5.97 | 12.54351522 | 1.21E-14 |
| 56 | Tbrg4 | transforming growth factor beta regulator 4 | 0.001 | 5.85 | 12.51422091 | 7.59E-52 |
| 27 | Gstcd | glutathione S-transferase C-terminal domain containing | 0.001 | 5.82 | 12.50680344 | 1.46E-54 |

**Table 3.** The top down-regulated mRNA after T antigen knockdown in lens tumor cells

| **Num** | **Symbol** | **Gene** | **JCVT** | **ShJCVT** | **log2(FC)** | **P value** |
| --- | --- | --- | --- | --- | --- | --- |
| 1 | Ptprs | protein tyrosine phosphatase receptor type S | 28.64 | 0.001 | -14.8057439 | 0 |
| 2 | Sptan1 | spectrin alpha, non-erythrocytic 1 | 23.31 | 0.001 | -14.5086614 | 0 |
| 3 | Epb41l1 | erythrocyte membrane protein band 4.1 like 1 | 14.56 | 0.001 | -13.8297227 | 0 |
| 4 | Psme2 | proteasome activator subunit 2 | 14.33 | 0.001 | -13.806751 | 7.92E-47 |
| 5 | Trim32 | tripartite motif containing 32 | 13.62 | 0.001 | -13.7334391 | 8.23E-176 |
| 6 | Myc | MYC proto-oncogene, bHLH transcription factor | 10.04 | 0.001 | -13.2934716 | 2.41E-56 |
| 7 | Dctd | dCMP deaminase | 8.83 | 0.001 | -13.1081977 | 5.14E-75 |
| 8 | Slu7 | SLU7 homolog, splicing factor | 8.73 | 0.001 | -13.0917659 | 1.90E-126 |
| 9 | Msantd3 | Myb/SANT DNA binding domain containing 3 | 8.5 | 0.001 | -13.0532471 | 1.88E-55 |
| 10 | Mef2a | myocyte enhancer factor 2A | 8.25 | 0.001 | -13.0101784 | 8.95E-90 |
| 11 | Sdf4 | stromal cell derived factor 4 | 8.16 | 0.001 | -12.9943534 | 8.21E-33 |
| 12 | Tmem33 | transmembrane protein 33 | 7.76 | 0.001 | -12.9218409 | 1.11E-195 |
| 13 | Nisch | nischarin | 7.75 | 0.001 | -12.9199806 | 2.41E-22 |
| 14 | Arhgap6 | Rho GTPase activating protein 6 | 7.6 | 0.001 | -12.8917837 | 2.80E-125 |
| 15 | Pmp22 | peripheral myelin protein 22 | 7.33 | 0.001 | -12.8395975 | 7.38E-55 |
| 16 | Mrpl2 | mitochondrial ribosomal protein L2 | 6.97 | 0.001 | -12.7669429 | 1.26E-28 |
| 17 | Rhoc | ras homolog family member C | 6.79 | 0.001 | -12.7291959 | 1.19E-16 |
| 18 | Ndufs5 | NADH:ubiquinone oxidoreductase subunit S5 | 6.77 | 0.001 | -12.7249401 | 2.39E-10 |
| 19 | Wdr45 | WD repeat domain 45 | 6.72 | 0.001 | -12.7142455 | 9.62E-45 |
| 20 | Flna | filamin A | 6.66 | 0.001 | -12.7013065 | 1.53E-23 |
| 21 | Usp28 | ubiquitin specific peptidase 28 | 6.46 | 0.001 | -12.6573184 | 2.68E-108 |
| 22 | Dclre1a | DNA cross-link repair 1A | 6.32 | 0.001 | -12.6257088 | 8.92E-105 |
| 23 | Rab43 | RAB43, member RAS oncogene family(human) | 6.24 | 0.001 | -12.6073303 | 1.80E-109 |
| 24 | Hspa2 | heat shock protein family A (Hsp70) member 2 | 6.18 | 0.001 | -12.5933911 | 4.64E-64 |
| 25 | Kirrel | kirre like nephrin family adhesion molecule 1 | 6.15 | 0.001 | -12.5863707 | 1.46E-54 |
| 26 | Pogk | pogo transposable element derived with KRAB domain | 5.92 | 0.001 | -12.5313815 | 4.77E-19 |
| 27 | Ube2e2 | ubiquitin conjugating enzyme E2 E2 | 5.89 | 0.001 | -12.5240519 | 3.77E-15 |
| 28 | Dnajb2 | DnaJ heat shock protein family (Hsp40) member B2 | 5.68 | 0.001 | -12.4716752 | 1.17E-42 |
| 29 | C1qtnf1 | C1q and TNF related 1 | 5.62 | 0.001 | -12.4563544 | 2.01E-58 |
| 30 | Ppm1m | protein phosphatase, Mg2+/Mn2+ dependent 1M | 5.61 | 0.001 | -12.4537851 | 9.18E-42 |
| 31 | Evi5 | ecotropic viral integration site 5 | 5.51 | 0.001 | -12.4278366 | 9.50E-19 |
| 32 | Mndal | myeloid nuclear differentiation antigen like | 5.49 | 0.001 | -12.4225904 | 2.97E-43 |
| 33 | Zmynd11 | zinc finger MYND-type containing 11 | 5.48 | 0.001 | -12.4199602 | 1.82E-84 |
| 34 | Zfp260 | zinc finger protein 260 | 5.32 | 0.001 | -12.3772105 | 1.46E-78 |
| 35 | Faf2 | Fas associated factor family member 2 | 5.31 | 0.001 | -12.3744961 | 3.81E-21 |
| 36 | Pygm | glycogen phosphorylase, muscle associated | 5.25 | 0.001 | -12.3581017 | 5.53E-62 |
| 37 | Plekha1 | pleckstrin homology domain containing A1 | 5.24 | 0.001 | -12.3553511 | 3.61E-71 |
| 38 | Ttc3 | tetratricopeptide repeat domain 3 | 5.24 | 0.001 | -12.3553511 | 6.32E-81 |
| 39 | Naa35 | N-alpha-acetyltransferase 35, NatC auxiliary subunit | 5.24 | 0.001 | -12.3553511 | 5.97E-14 |
| 40 | Cacng7 | calcium voltage-gated channel auxiliary subunit gamma 7 | 5.13 | 0.001 | -12.3247431 | 7.92E-47 |
| 41 | Camsap3 | calmodulin regulated spectrin associated protein family member 3 | 5.06 | 0.001 | -12.3049217 | 4.86E-80 |
| 42 | Ctsa | cathepsin A | 5.01 | 0.001 | -12.2905949 | 1.50E-14 |
| 43 | Ecm1 | extracellular matrix protein 1 | 4.82 | 0.001 | -12.2348174 | 6.91E-38 |
| 44 | Mpp5 | membrane palmitoylated protein 5 | 4.81 | 0.001 | -12.2318212 | 4.00E-107 |
| 45 | Rfx5 | regulatory factor X5 | 4.76 | 0.001 | -12.2167459 | 8.23E-82 |
| 46 | Nrf1 | nuclear respiratory factor 1 | 4.73 | 0.001 | -12.2076245 | 4.02E-30 |
| 47 | Aim2 | absent in melanoma 2 | 4.64 | 0.001 | -12.1799091 | 5.75E-54 |
| 48 | Thoc7 | THO complex 7 | 4.43 | 0.001 | -12.113091 | 2.99E-14 |
| 49 | Plk4 | polo like kinase 4 | 4.42 | 0.001 | -12.1098307 | 4.29E-61 |
| 50 | Fggy | FGGY carbohydrate kinase domain containing | 4.38 | 0.001 | -12.0967152 | 1.33E-34 |
| 51 | Sp2 | Sp2 transcription factor | 4.37 | 0.001 | -12.0934176 | 1.76E-52 |
| 52 | Rnasel | ribonuclease L | 4.34 | 0.001 | -12.0834793 | 1.76E-52 |
| 53 | Ly6a | lymphocyte antigen 6 complex, locus A | 4.33 | 0.001 | -12.0801513 | 7.52E-15 |
| 54 | Uck1 | uridine-cytidine kinase 1 | 4.32 | 0.001 | -12.0768156 | 1.50E-14 |
| 55 | Zfp966 | zinc finger protein 966 | 4.21 | 0.001 | -12.0396045 | 5.40E-51 |
| 56 | Tmem94 | transmembrane protein 94 | 4.15 | 0.001 | -12.0188956 | 3.48E-89 |

**Table 4.** The top mRNA-related signal pathways after T antigen knockdown in lens tumor cells

| **N Num** | **Pathway** | **DEGs genes** | **P value** |
| --- | --- | --- | --- |
| 1 | [Systemic lupus erythematosus](#说明文件!gene1) | 66 (2.61%) | <0.00001 |
| 2 | [Viral carcinogenesis](#说明文件!gene2) | 143 (5.66%) | <0.00001 |
| 3 | [Alcoholism](#说明文件!gene3) | 111 (4.39%) | <0.00001 |
| 4 | [Focal adhesion](#说明文件!gene4) | 118 (4.67%) | 0.000001 |
| 5 | [Choline metabolism in cancer](#说明文件!gene5) | 68 (2.69%) | 0.000003 |
| 6 | [MicroRNAs in cancer](#说明文件!gene6) | 91 (3.6%) | 0.000008 |
| 7 | [p53 signaling pathway](#说明文件!gene7) | 52 (2.06%) | 0.000009 |
| 8 | [Phospholipase D signaling pathway](#说明文件!gene8) | 93 (3.68%) | 0.000009 |
| 9 | [Biosynthesis of secondary metabolites](#说明文件!gene9) | 177 (7%) | 0.00001 |
| 10 | [TNF signaling pathway](#说明文件!gene10) | 68 (2.69%) | 0.000019 |
| 11 | [Biosynthesis of antibiotics](#说明文件!gene11) | 106 (4.19%) | 0.000042 |
| 12 | [Prostate cancer](#说明文件!gene12) | 67 (2.65%) | 0.000049 |
| 13 | [Lysine degradation](#说明文件!gene13) | 38 (1.5%) | 0.000057 |
| 14 | [Cell cycle](#说明文件!gene14) | 67 (2.65%) | 0.000103 |
| 15 | [Insulin resistance](#说明文件!gene15) | 65 (2.57%) | 0.000146 |
| 16 | [Pyrimidine metabolism](#说明文件!gene16) | 54 (2.14%) | 0.000186 |
| 17 | [AGE-RAGE signaling pathway in diabetic complications](#说明文件!gene17) | 64 (2.53%) | 0.000192 |
| 18 | [Metabolic pathways](#说明文件!gene18) | 476 (18.83%) | 0.000212 |
| 19 | [Ribosome biogenesis in eukaryotes](#说明文件!gene19) | 38 (1.5%) | 0.000224 |
| 20 | [Fc gamma R-mediated phagocytosis](#说明文件!gene20) | 61 (2.41%) | 0.000265 |
| 21 | [RNA transport](#说明文件!gene21) | 76 (3.01%) | 0.000282 |
| 22 | [Toll-like receptor signaling pathway](#说明文件!gene22) | 60 (2.37%) | 0.000285 |
| 23 | [PI3K-Akt signaling pathway](#说明文件!gene23) | 161 (6.37%) | 0.000327 |
| 24 | [Glucagon signaling pathway](#说明文件!gene24) | 63 (2.49%) | 0.000491 |
| 25 | [Insulin signaling pathway](#说明文件!gene25) | 84 (3.32%) | 0.000638 |
| 26 | [Sphingolipid signaling pathway](#说明文件!gene26) | 65 (2.57%) | 0.00066 |
| 27 | [Biosynthesis of amino acids](#说明文件!gene27) | 44 (1.74%) | 0.000719 |
| 28 | [DNA replication](#说明文件!gene28) | 21 (0.83%) | 0.000735 |
| 29 | [Influenza A](#说明文件!gene29) | 84 (3.32%) | 0.00074 |
| 30 | [Small cell lung cancer](#说明文件!gene30) | 51 (2.02%) | 0.000825 |
| 31 | [Ras signaling pathway](#说明文件!gene31) | 124 (4.91%) | 0.00129 |
| 32 | [Hepatitis B](#说明文件!gene32) | 80 (3.16%) | 0.001446 |
| 33 | [HIF-1 signaling pathway](#说明文件!gene33) | 73 (2.89%) | 0.001504 |
| 34 | [Tuberculosis](#说明文件!gene34) | 88 (3.48%) | 0.001646 |
| 35 | [Acute myeloid leukemia](#说明文件!gene35) | 44 (1.74%) | 0.001696 |
| 36 | [FoxO signaling pathway](#说明文件!gene36) | 73 (2.89%) | 0.001742 |
| 37 | [HTLV-I infection](#说明文件!gene37) | 125 (4.94%) | 0.001749 |
| 38 | [Rap1 signaling pathway](#说明文件!gene38) | 119 (4.71%) | 0.001753 |
| 39 | [Fructose and mannose metabolism](#说明文件!gene39) | 27 (1.07%) | 0.001774 |
| 40 | [Pancreatic cancer](#说明文件!gene40) | 48 (1.9%) | 0.002042 |
| 41 | [RNA degradation](#说明文件!gene41) | 39 (1.54%) | 0.002206 |
| 42 | [AMPK signaling pathway](#说明文件!gene42) | 67 (2.65%) | 0.002232 |
| 43 | [VEGF signaling pathway](#说明文件!gene43) | 40 (1.58%) | 0.00228 |
| 44 | [Glioma](#说明文件!gene44) | 44 (1.74%) | 0.003053 |
| 45 | [Herpes simplex infection](#说明文件!gene45) | 93 (3.68%) | 0.003836 |
| 46 | [Longevity regulating pathway - mammal](#说明文件!gene46) | 55 (2.18%) | 0.003916 |
| 47 | [Spliceosome](#说明文件!gene47) | 54 (2.14%) | 0.003916 |

**Table 5.** The top up-regulated miRNAs after T antigen knockdown in lens tumor cells

| **Num** | **ID** | **JCV T TPM** | **ShJCV T TPM** | **Log2(fc)** | **P value** |
| --- | --- | --- | --- | --- | --- |
| 1 | miR-4877-y | 0.01 | 48.3367 | 12.23890327 | 2.77E-52 |
| 2 | miR-1249-y | 0.01 | 5.0967 | 8.993419626 | 1.57E-06 |
| 3 | miR-139-y | 0.01 | 4.2747 | 8.739679364 | 2.12E-05 |
| 4 | miR-7-x | 0.01 | 3.617 | 8.498649788 | 0.000145836 |
| 5 | miR-458-y | 0.01 | 3.4526 | 8.431539391 | 0.000276113 |
| 6 | miR-365-x | 0.01 | 3.2882 | 8.361154241 | 0.000521183 |
| 7 | miR-532-y | 0.01 | 2.6306 | 8.039248084 | 0.003427498 |
| 8 | miR-205-x | 0.01 | 2.4662 | 7.946145992 | 0.006362167 |
| 9 | miR-6390-x | 0.01 | 2.4662 | 7.946145992 | 0.006362167 |
| 10 | miR-7669-y | 0.01 | 2.3017 | 7.846555997 | 0.006362167 |
| 11 | miR-365-y | 1.9241 | 13.9749 | 2.860582273 | 3.22E-09 |
| 12 | miR-1599-y | 0.6414 | 4.6035 | 2.843434888 | 0.000916869 |
| 13 | miR-3470-x | 0.6414 | 4.4391 | 2.79097095 | 0.001553398 |
| 14 | miR-702-y | 0.6414 | 4.2747 | 2.736526916 | 0.001553398 |
| 15 | miR-5099-y | 21.1651 | 140.4065 | 2.729850515 | 3.01E-78 |
| 16 | miR-194-x | 0.6414 | 4.1103 | 2.679947437 | 0.00261354 |
| 17 | miR-362-x | 0.6414 | 4.1103 | 2.679947437 | 0.00261354 |
| 18 | miR-1946-z | 0.6414 | 3.7814 | 2.559624208 | 0.007226519 |
| 19 | miR-34-y | 1.9241 | 10.3579 | 2.428475849 | 2.10E-06 |
| 20 | miR-21-x | 1500.8009 | 7234.3868 | 2.269138138 | <0.00001 |
| 21 | miR-3473-y | 1.9241 | 9.207 | 2.258547365 | 2.30E-05 |
| 22 | miR-1946-x | 1.9241 | 8.3849 | 2.123609797 | 0.000144544 |

**Table 6.** The top down-regulated miRNAs after T antigen knockdown in lens tumor cells

| **Num** | **ID** | **JCVT TPM** | **ShJCVT TPM** | **Log2(fc)** | **P value** |
| --- | --- | --- | --- | --- | --- |
| 1 | miR-460-x | 44.2544 | 0.01 | -12.1116052 | 8.37E-50 |
| 2 | miR-8402-y | 18.9204 | 0.01 | -10.8857269 | 1.91E-21 |
| 3 | miR-8410-y | 12.8274 | 0.01 | -10.3250131 | 1.50E-14 |
| 4 | miR-5119-y | 9.9412 | 0.01 | -9.9572762 | 1.50E-11 |
| 5 | miR-9231-x | 8.6585 | 0.01 | -9.7579733 | 4.77E-10 |
| 6 | miR-96-x | 8.6585 | 0.01 | -9.7579733 | 4.77E-10 |
| 7 | miR-8427-y | 8.0171 | 0.01 | -9.64693666 | 1.90E-09 |
| 8 | miR-2566-x | 6.093 | 0.01 | -9.25100893 | 2.41E-07 |
| 9 | miR-766-x | 5.7723 | 0.01 | -9.17300247 | 9.64E-07 |
| 10 | miR-1336-x | 5.4516 | 0.01 | -9.0905359 | 1.93E-06 |
| 11 | miR-190-x | 4.8103 | 0.01 | -8.90998306 | 7.69E-06 |
| 12 | miR-8962-y | 4.8103 | 0.01 | -8.90998306 | 7.69E-06 |
| 13 | miR-2209-y | 3.8482 | 0.01 | -8.58803997 | 0.000122627 |
| 14 | miR-5107-y | 3.8482 | 0.01 | -8.58803997 | 0.000122627 |
| 15 | miR-2763-x | 3.5275 | 0.01 | -8.46250227 | 0.000122627 |
| 16 | miR-4448-y | 3.5275 | 0.01 | -8.46250227 | 0.000122627 |
| 17 | miR-4561-y | 3.5275 | 0.01 | -8.46250227 | 0.000122627 |
| 18 | miR-430-x | 3.2068 | 0.01 | -8.32499057 | 0.000489894 |
| 19 | miR-8358-y | 3.2068 | 0.01 | -8.32499057 | 0.000489894 |
| 20 | miR-490-y | 2.8862 | 0.01 | -8.17302747 | 0.00097925 |
| 21 | miR-6582-y | 2.8862 | 0.01 | -8.17302747 | 0.00097925 |
| 22 | miR-153-x | 2.5655 | 0.01 | -8.00309622 | 0.001957522 |
| 23 | miR-2285-y | 2.5655 | 0.01 | -8.00309622 | 0.001957522 |
| 24 | miR-4503-x | 2.5655 | 0.01 | -8.00309622 | 0.001957522 |
| 25 | miR-6916-x | 2.5655 | 0.01 | -8.00309622 | 0.001957522 |
| 26 | miR-7185-y | 2.5655 | 0.01 | -8.00309622 | 0.001957522 |
| 27 | miR-7292-x | 2.5655 | 0.01 | -8.00309622 | 0.001957522 |
| 28 | miR-1537-y | 2.2448 | 0.01 | -7.8104431 | 0.003913285 |
| 29 | miR-2226-x | 2.2448 | 0.01 | -7.8104431 | 0.003913285 |
| 30 | miR-2753-y | 2.2448 | 0.01 | -7.8104431 | 0.003913285 |
| 31 | miR-342-x | 2.2448 | 0.01 | -7.8104431 | 0.003913285 |
| 32 | miR-3897-y | 2.2448 | 0.01 | -7.8104431 | 0.003913285 |
| 33 | miR-4001-x | 2.2448 | 0.01 | -7.8104431 | 0.003913285 |
| 34 | miR-4826-x | 2.2448 | 0.01 | -7.8104431 | 0.003913285 |
| 35 | miR-4913-y | 2.2448 | 0.01 | -7.8104431 | 0.003913285 |
| 36 | miR-548-x | 2.2448 | 0.01 | -7.8104431 | 0.003913285 |
| 37 | miR-873-y | 2.2448 | 0.01 | -7.8104431 | 0.003913285 |
| 38 | miR-4819-x | 1.9241 | 0.01 | -7.58803997 | 0.01564141 |
| 39 | miR-9546-x | 1.9241 | 0.01 | -7.58803997 | 0.01564141 |
| 40 | miR-1187-y | 1.6034 | 0.01 | -7.32499057 | 0.03127344 |
| 41 | miR-1326-y | 1.6034 | 0.01 | -7.32499057 | 0.03127344 |
| 42 | miR-430-y | 1.6034 | 0.01 | -7.32499057 | 0.03127344 |
| 43 | miR-507-y | 1.6034 | 0.01 | -7.32499057 | 0.03127344 |
| 44 | miR-544-y | 1.6034 | 0.01 | -7.32499057 | 0.03127344 |
| 45 | miR-5551-x | 1.6034 | 0.01 | -7.32499057 | 0.03127344 |
| 46 | miR-7386-y | 1.6034 | 0.01 | -7.32499057 | 0.03127344 |
| 47 | miR-7469-x | 1.6034 | 0.01 | -7.32499057 | 0.03127344 |
| 48 | miR-9571-y | 1.6034 | 0.01 | -7.32499057 | 0.03127344 |
| 49 | miR-8940-y | 134.6873 | 1.8085 | -6.21867641 | 3.35E-151 |
| 50 | miR-9464-y | 44.8958 | 0.6576 | -6.09322638 | 2.72E-51 |
| 51 | miR-96-y | 49.3853 | 0.8221 | -5.90862397 | 2.41E-56 |
| 52 | miR-7194-y | 83.3778 | 1.6441 | -5.66429335 | 8.82E-95 |
| 53 | miR-9177-y | 33.9925 | 0.9865 | -5.10675361 | 1.12E-39 |
| 54 | miR-3242-x | 16.6756 | 0.4932 | -5.07942206 | 1.20E-19 |
| 55 | miR-8228-y | 129.5563 | 3.9458 | -5.03711753 | 5.25E-148 |
| 56 | miR-2247-x | 7.3757 | 0.4932 | -3.90253527 | 5.88E-08 |
| 57 | miR-4961-y | 3.8482 | 0.3288 | -3.54890158 | 0.000521183 |
| 58 | miR-4982-y | 3.5275 | 0.3288 | -3.42336388 | 0.000980413 |
| 59 | miR-6747-y | 3.5275 | 0.3288 | -3.42336388 | 0.000980413 |
| 60 | miR-1192-x | 4.8103 | 0.4932 | -3.28588217 | 4.04E-05 |
| 61 | miR-252-y | 2.8862 | 0.3288 | -3.13388907 | 0.006362167 |
| 62 | miR-962-x | 3.2068 | 0.4932 | -2.70088967 | 0.007406097 |
| 63 | miR-9-y | 3.2068 | 0.4932 | -2.70088967 | 0.007406097 |
| 64 | miR-210-y | 3.5275 | 0.6576 | -2.42336388 | 0.00419502 |
| 65 | miR-7114-y | 17.3169 | 3.4526 | -2.32642558 | 7.62E-11 |
| 66 | miR-235-y | 3.2068 | 0.6576 | -2.28585218 | 0.007406097 |
| 67 | miR-203-y | 4.1689 | 0.9865 | -2.07927581 | 0.007226519 |

**Table 7.** The top up-regulated lncRNAs after T antigen knockdown in lens tumor cells

| **Num** | **Symbol** | **JCVT** | **ShJCVT** | **log2(FC)** | **P value** |
| --- | --- | --- | --- | --- | --- |
| 1 | Dleu2 | 0.001 | 3 | 11.55074679 | 5.80E-48 |
| 2 | Snhg6 | 0.001 | 2.43 | 11.2467406 | 5.78E-06 |
| 3 | Snhg17 | 0.001 | 2.17 | 11.08347933 | 5.78E-06 |
| 4 | Gm17768 | 0.001 | 1.96 | 10.93663794 | 0.000980413 |
| 5 | Dleu2 | 0.001 | 1.25 | 10.28771238 | 0.000521183 |
| 6 | Gm12784 | 0.001 | 1.05 | 10.03617361 | 0.003427498 |
| 7 | 1810058I24Rik | 0.02 | 2.38 | 6.894817763 | 2.41E-13 |
| 8 | AC118476.2 | 0.05 | 1.02 | 4.350497247 | 6.85E-08 |
| 9 | CR974585.1 | 0.07 | 1.17 | 4.063009798 | 4.72E-06 |
| 10 | Jpx | 0.19 | 2.48 | 3.706268797 | 1.11E-06 |
| 11 | AC145731.3 | 0.14 | 1.69 | 3.593524514 | 8.12E-08 |
| 12 | AC154864.1 | 0.23 | 2.3 | 3.321928095 | 3.19E-07 |
| 13 | AC122346.1 | 0.29 | 1.97 | 2.764070824 | 5.33E-20 |
| 14 | 4930558J18Rik | 0.26 | 1.74 | 2.742503778 | 2.21E-10 |
| 15 | AC164662.1 | 0.29 | 1.81 | 2.641864892 | 0.000916869 |
| 16 | AL513022.1 | 0.96 | 5.79 | 2.592457037 | 1.35E-31 |
| 17 | Snhg1 | 0.8 | 4.64 | 2.5360529 | 3.04E-09 |
| 18 | Gm10505 | 0.4 | 2.28 | 2.510961919 | 1.22E-09 |
| 19 | Gm26870 | 0.37 | 2.07 | 2.484033592 | 7.52E-07 |
| 20 | 2410012E07Rik | 1.74 | 8.78 | 2.335133634 | 2.29E-27 |
| 21 | Gm3764 | 0.33 | 1.66 | 2.330645312 | 6.99E-05 |
| 22 | Snhg8 | 2.19 | 10.74 | 2.293991218 | 9.75E-12 |
| 23 | Ftx | 0.22 | 1.02 | 2.212993723 | 2.10E-06 |
| 24 | Gm5113 | 0.52 | 2.33 | 2.163746427 | 4.68E-25 |
| 25 | AC160063.1 | 0.27 | 1.17 | 2.115477217 | 0.002509385 |
| 26 | Gm14005 | 0.79 | 3.2 | 2.018147347 | 2.30E-05 |

**Table 8.** The top down-regulated lncRNAs after T antigen knockdown in lens tumor cells

| **Num** | **Symbol** | **JCVT fpkm** | **ShJCVT fpkm** | **log2(FC)** | **P value** |
| --- | --- | --- | --- | --- | --- |
| 1 | Snhg8 | 5.48 | 0.001 | -12.4199602 | 7.54E-12 |
| 2 | 4933433G15Rik | 1.56 | 0.01 | -7.28540222 | 2.41E-07 |
| 3 | Malat1 | 219.18 | 4.52 | -5.59964958 | <0.00001 |
| 4 | Gm12056 | 12.05 | 0.29 | -5.37683644 | 2.17E-61 |
| 5 | B430319H21Rik | 2.09 | 0.11 | -4.24792751 | 1.11E-05 |
| 6 | Carmn | 3.08 | 0.31 | -3.31259023 | 5.28E-09 |
| 7 | 4930523C07Rik | 12.95 | 1.91 | -2.76130755 | 3.06E-152 |
| 8 | AC162934.1 | 2 | 0.3 | -2.73696559 | 2.48E-06 |
| 9 | C78283 | 1.81 | 0.29 | -2.64186489 | 0.000279321 |
| 10 | Gm35021 | 1.8 | 0.3 | -2.5849625 | 2.50E-09 |
| 11 | Gm16001 | 4.39 | 0.75 | -2.54925844 | 2.79E-09 |
| 12 | AC122313.3 | 2.87 | 0.53 | -2.43698647 | 1.58E-07 |
| 13 | CT009733.2 | 1.18 | 0.22 | -2.42321143 | 6.36E-11 |
| 14 | 2210011K15Rik | 1.35 | 0.26 | -2.37637588 | 4.30E-05 |
| 15 | BC030343 | 1.22 | 0.25 | -2.28688115 | 3.48E-06 |
| 16 | Gm3830 | 1.1 | 0.26 | -2.08092 | 0.000829453 |
| 17 | Snhg12 | 4.84 | 1.19 | -2.02404547 | 2.54E-07 |

**Table 9.** The top metabolite-related signal pathways after T antigen knockdown in lens tumor cells

| **Num** | **Pathway** | **JCVT/hJCVT** | **P value** |
| --- | --- | --- | --- |
| 1 | Monobactam biosynthesis | 9 | 0.00053012 |
| 2 | Protein digestion and absorption | 18 | 0.001517864 |
| 3 | Biosynthesis of antibiotics | 41 | 0.002506747 |
| 4 | Aminoacyl-tRNA biosynthesis | 13 | 0.005676289 |
| 5 | Biosynthesis of amino acids | 26 | 0.01051312 |
| 6 | Longevity regulating pathway - mammal | 5 | 0.01549513 |
| 7 | Inflammatory mediator regulation of TRP channels | 5 | 0.01549513 |
| 8 | Glycine, serine and threonine metabolism | 14 | 0.02633682 |
| 9 | Renin secretion | 6 | 0.02953491 |
| 10 | Salivary secretion | 6 | 0.02953491 |
| 11 | Gastric acid secretion | 6 | 0.02953491 |
| 12 | Pancreatic secretion | 6 | 0.02953491 |
| 13 | Degradation of aromatic compounds | 20 | 0.03321145 |
| 14 | Platelet activation | 4 | 0.03582072 |
| 15 | Cholinergic synapse | 4 | 0.03582072 |
| 16 | Olfactory transduction | 4 | 0.03582072 |
| 17 | AMPK signaling pathway | 7 | 0.04057589 |
| 18 | Proximal tubule bicarbonate reclamation | 7 | 0.04057589 |
| 19 | Arginine and proline metabolism | 15 | 0.04585785 |
| 20 | Pyruvate metabolism | 8 | 0.04899864 |

**Table 10.** The top up-regulated proteins after T antigen knockdown in lens tumor cells

| **Num** | **Symbol** | **Gene** | **ShJCVT/JCVT** | **p Value** |
| --- | --- | --- | --- | --- |
| 1 | Bckdhb | branched chain keto acid dehydrogenase E1 subunit beta | 37.72 | 0.02809156 |
| 2 | Tacc3 | transforming acidic coiled-coil containing protein 3 | 34.31 | 0.01256062 |
| 3 | Zzef1 | zinc finger ZZ-type and EF-hand domain containing 1 | 24.76 | 0.03421757 |
| 4 | Plin2 | perilipin 2 | 24.25 | 0.03508748 |
| 5 | Cab39l | calcium binding protein 39 like | 21.79 | 0.02018604 |
| 6 | Elmo2 | engulfment and cell motility 2 | 20.53 | 0.00013041 |
| 7 | Fgfr1op | FGFR1 oncogene partner | 19.22 | 0.000449186 |
| 8 | Lcp1 | lymphocyte cytosolic protein 1 | 18.56 | 0.002412462 |
| 9 | Ccdc137 | coiled-coil domain containing 137 | 17.79 | 0.003327602 |
| 10 | Lnpk | lunapark, ER junction formation factor | 17.38 | 0.002183018 |
| 11 | Arhgap22 | Rho GTPase activating protein 22 | 17.35 | 0.001726332 |
| 12 | Nsun5 | NOP2/Sun RNA methyltransferase 5 | 17.35 | 0.03995623 |
| 13 | Slc25a32 | solute carrier family 25 member 32 | 17.02 | 0.01526827 |
| 14 | Myo10 | myosin X | 15.26 | 0.008331147 |
| 15 | Kif20a | kinesin family member 20A | 15.18 | 0.04847868 |
| 16 | Kif22 | kinesin family member 22 | 14.83 | 0.004279496 |
| 17 | Rbl1 | https://www.ncbi.nlm.nih.gov/gene/?term=Rbl1 | 14.42 | 0.003021872 |
| 18 | Prepl | prolyl endopeptidase like | 14.3 | 0.003945316 |
| 19 | Atm | ATM serine/threonine kinase | 13.23 | 0.04349202 |
| 20 | Lars2 | leucyl-tRNA synthetase 2, mitochondrial | 13.21 | 9.74E-05 |
| 21 | Aatf | apoptosis antagonizing transcription factor | 13.1 | 0.002082746 |
| 22 | Dcaf13 | DDB1 and CUL4 associated factor 13 | 13.07 | 0.000202424 |
| 23 | Ascc3 | activating signal cointegrator 1 complex subunit 3 | 12.95 | 0.007281793 |
| 24 | Med24 | mediator complex subunit 24 | 12.59 | 0.002533956 |
| 25 | Espl1 | extra spindle pole bodies like 1, separase | 12.47 | 0.000712799 |
| 26 | Parvb | parvin beta | 12.34 | 0.02536614 |
| 27 | Akap2 | https://www.ncbi.nlm.nih.gov/gene/?term=Akap2 | 12.33 | 0.001039333 |
| 28 | Urb2 | URB2 ribosome biogenesis homolog | 11.92 | 0.0115204 |
| 29 | Tagln | transgelin | 11.85 | 2.66E-05 |
| 30 | Bgn | biglycan | 11.79 | 0.001859917 |
| 31 | Commd5 | COMM domain containing 5 | 11.62 | 0.03652918 |
| 32 | Pex5 | peroxisomal biogenesis factor 5 | 11.22 | 0.04831508 |
| 33 | Sun1 | Sad1 and UNC84 domain containing 1 | 11.15 | 0.0476518 |
| 34 | Fblim1 | filamin binding LIM protein 1 | 11.13 | 0.004130766 |
| 35 | Orc1 | origin recognition complex subunit 1 | 11.03 | 3.28E-05 |
| 36 | Lgals9 | galectin 9 | 10.7 | 0.009869054 |
| 37 | Trmt10c | tRNA methyltransferase 10C, mitochondrial RNase P subunit | 10.66 | 0.006496282 |
| 38 | Abcb1b | ATP-binding cassette, sub-family B (MDR/TAP), member 1B | 10.42 | 0.003429423 |
| 39 | Anln | anillin actin binding protein | 10.12 | 6.56E-06 |

**Table 11.** The top down-regulated proteins after T antigen knockdown in lens tumor cells

| **Num** | **Symbol** | **Gene** | **ShJCVT/JCVT** | **p Value** |
| --- | --- | --- | --- | --- |
| 1 | Ddx58 | DExD/H-box helicase 58 | 0.01417 | 0.000291 |
| 2 | Scrn1 | secernin 1 | 0.0331 | 0.0193987 |
| 3 | Naprt | nicotinate phosphoribosyltransferase | 0.04829 | 0.0031831 |
| 4 | Samd9l | sterile alpha motif domain containing 9 like | 0.08904 | 0.0003384 |
| 5 | Nqo1 | NAD(P)H quinone dehydrogenase 1 | 0.08908 | 8.82E-05 |
| 6 | Pdgfra | platelet derived growth factor receptor alpha | 0.1083 | 0.0054324 |
| 7 | Bcl2 | BCL2 apoptosis regulator | 0.1235 | 0.0002351 |
| 8 | Glg1 | golgi glycoprotein 1 | 0.1236 | 0.0018557 |
| 9 | Emilin1 | elastin microfibril interfacer 1 | 0.1336 | 0.0144433 |
| 10 | Col6a1 | collagen type VI alpha 1 chain | 0.1415 | 0.0137663 |
| 11 | Hspg2 | heparan sulfate proteoglycan 2 | 0.1509 | 0.0009172 |
| 12 | Fam129a | family with sequence similarity 129 member A | 0.1555 | 0.0001579 |
| 13 | Prkar1a | protein kinase cAMP-dependent type I regulatory subunit alpha | 0.1669 | 0.0061616 |
| 14 | Ifi35 | interferon induced protein 35 | 0.1737 | 0.0347299 |
| 15 | Eml2 | EMAP like 2 | 0.1768 | 0.0044588 |
| 16 | Baiap2 | BAR/IMD domain containing adaptor protein 2 | 0.1778 | 5.81E-05 |
| 17 | Fam98c | family with sequence similarity 98 member C | 0.1884 | 0.0187212 |
| 18 | Rps6ka1 | ribosomal protein S6 kinase A1 | 0.2124 | 0.015886 |
| 19 | Pdp1 | pyruvate dehyrogenase phosphatase catalytic subunit 1 | 0.22 | 0.0300462 |
| 20 | Rnf213 | ring finger protein 213 | 0.2289 | 1.03E-12 |
| 21 | Nfkb2 | nuclear factor kappa B subunit 2 | 0.238 | 0.0150912 |
| 22 | F11r | F11 receptor | 0.2423 | 0.0270059 |
| 23 | Stat1 | signal transducer and activator of transcription 1 | 0.2656 | 0.0001146 |
| 24 | Ubxn7 | UBX domain protein 7 | 0.2798 | 0.0045471 |
| 25 | Mov10 | Mov10 RISC complex RNA helicase | 0.2821 | 0.0129194 |
| 26 | Prune2 | prune homolog 2 with BCH domain | 0.2846 | 0.0363062 |
| 27 | Loxl1 | lysyl oxidase like 1 | 0.2908 | 0.0252918 |
| 28 | Sphk1 | sphingosine kinase 1 | 0.2996 | 0.0107347 |
| 29 | S100a16 | S100 calcium binding protein A16 | 0.3048 | 0.0388132 |
| 30 | Pdcd4 | programmed cell death 4 | 0.3352 | 5.19E-06 |
| 31 | Psme2 | proteasome activator subunit 2 | 0.361 | 0.0195727 |
| 32 | Tnpo2 | transportin 2 | 0.3678 | 0.0090188 |
| 33 | Lrpap1 | LDL receptor related protein associated protein 1 | 0.3706 | 0.0281906 |
| 34 | Fscn1 | fascin actin-bundling protein 1 | 0.3726 | 0.0132751 |
| 35 | Edc3 | enhancer of mRNA decapping 3 | 0.3811 | 0.0109113 |
| 36 | Psmb8 | proteasome 20S subunit beta 8 | 0.3847 | 0.0085183 |
| 37 | Gstm1 | glutathione S-transferase mu 1 | 0.3896 | 0.0176187 |

**Table 12.** The top protein-related pathways after T antigen knockdown in lens tumor cells

| **Num** | **Description** | **Gene Ratio** | **p value** |
| --- | --- | --- | --- |
| 1 | [Ribosome biogenesis in eukaryotes](#说明文件!gene1) | 32 (4.24%) | 0.000002 |
| 2 | [Citrate cycle (TCA cycle)](#说明文件!gene2) | 19 (2.52%) | 0.000004 |
| 3 | [2-Oxocarboxylic acid metabolism](#说明文件!gene3) | 13 (1.72%) | 0.000078 |
| 4 | [Aminoacyl-tRNA biosynthesis](#说明文件!gene4) | 19 (2.52%) | 0.001062 |
| 5 | [Carbon metabolism](#说明文件!gene5) | 39 (5.17%) | 0.00208 |
| 6 | [N-Glycan biosynthesis](#说明文件!gene6) | 11 (1.46%) | 0.004363 |
| 7 | [Cell adhesion molecules (CAMs)](#说明文件!gene7) | 9 (1.19%) | 0.005493 |
| 8 | [Other types of O-glycan biosynthesis](#说明文件!gene8) | 5 (0.66%) | 0.007043 |
| 9 | [Protein digestion and absorption](#说明文件!gene9) | 11 (1.46%) | 0.009254 |
| 10 | [Phenylalanine metabolism](#说明文件!gene10) | 4 (0.53%) | 0.012422 |
| 11 | [Phenylalanine, tyrosine and tryptophan biosynthesis](#说明文件!gene11) | 2 (0.26%) | 0.012548 |
| 12 | [Biosynthesis of antibiotics](#说明文件!gene12) | 59 (7.81%) | 0.014332 |
| 13 | [Fat digestion and absorption](#说明文件!gene13) | 5 (0.66%) | 0.014719 |
| 14 | [RNA transport](#说明文件!gene14) | 46 (6.09%) | 0.020794 |
| 15 | [Microbial metabolism in diverse environments](#说明文件!gene15) | 44 (5.83%) | 0.03078 |
| 16 | [Biosynthesis of secondary metabolites](#说明文件!gene16) | 80 (10.6%) | 0.031468 |
| 17 | [Non-homologous end-joining](#说明文件!gene17) | 5 (0.66%) | 0.034284 |
| 18 | [Glyoxylate and dicarboxylate metabolism](#说明文件!gene18) | 8 (1.06%) | 0.036286 |
| 19 | [Steroid biosynthesis](#说明文件!gene19) | 6 (0.79%) | 0.036966 |
| 20 | [Biosynthesis of amino acids](#说明文件!gene20) | 26 (3.44%) | 0.039337 |
| 21 | [Porphyrin and chlorophyll metabolism](#说明文件!gene21) | 8 (1.06%) | 0.048826 |
| 22 | [Mismatch repair](#说明文件!gene22) | 8 (1.06%) | 0.048826 |
| 23 | [ECM-receptor interaction](#说明文件!gene23) | 11 (1.46%) | 0.049043 |

**Table 13.** The proteins from 25Kd band binding to T antigen in lens tumor cells

| **Num** | **Description** | **Score** | **Coverage** | **AA** | **MW [kDa]** | **pI** |
| --- | --- | --- | --- | --- | --- | --- |
| 1 | keratin, type I cytoskeletal 10 | 28.76 | 11.41 | 561 | 57 | 5.07 |
| 2 | 60S ribosomal protein L19 isoform 2 | 22.33 | 25.77 | 194 | 23.2 | 11.47 |
| 3 | apolipoprotein A-I preproprotein | 20.92 | 28.03 | 264 | 30.6 | 5.73 |
| 4 | keratin, type I cytoskeletal 15 | 18.44 | 9.87 | 456 | 49.5 | 4.86 |
| 5 | keratin, type II cytoskeletal 1 | 17.25 | 5.97 | 637 | 65.6 | 8.15 |
| 6 | keratin, type I cytoskeletal 14 isoform 1 | 15.85 | 8.26 | 484 | 52.8 | 5.17 |
| 7 | 60S ribosomal protein L14 | 15.21 | 17.05 | 217 | 23.5 | 11.02 |
| 8 | keratin, type II cytoskeletal 2 epidermal isoform X1 | 13.22 | 6.27 | 654 | 66.6 | 7.39 |
| 9 | keratin, type II cytoskeletal 5 | 10.47 | 7.07 | 580 | 61.7 | 7.75 |
| 10 | 60S ribosomal protein L18 isoform X1 | 10.31 | 26.47 | 136 | 15.2 | 11.81 |
| 11 | peroxiredoxin-1 | 9.17 | 9.55 | 199 | 22.2 | 8.12 |
| 12 | keratin, type II cytoskeletal 2 oral | 8.76 | 3.03 | 594 | 62.8 | 8.43 |
| 13 | keratin Kb40 isoform X2 | 8.27 | 1.37 | 1025 | 107.9 | 7.3 |
| 14 | keratin, type II cytoskeletal 79 | 7.88 | 5.84 | 531 | 57.5 | 7.69 |
| 15 | uncharacterized protein LOC75471 | 7.28 | 13.43 | 216 | 24.3 | 7.97 |
| 16 | 60S ribosomal protein L10-like | 6.84 | 9.35 | 214 | 24.5 | 10.11 |
| 17 | ATP synthase F(0) complex subunit B1, mitochondrial isoform 2 | 6.8 | 11.26 | 231 | 26.6 | 8.9 |
| 18 | triosephosphate isomerase | 6.54 | 10.03 | 299 | 32.2 | 5.74 |
| 19 | 60S ribosomal protein L29 | 4.72 | 11.88 | 160 | 17.6 | 11.84 |
| 20 | myeloid-associated differentiation marker | 4.29 | 8.44 | 320 | 35.3 | 8.31 |
| 21 | rho-related GTP-binding protein RhoC precursor | 4.26 | 9.84 | 193 | 22 | 6.58 |
| 22 | 60S ribosomal protein L13a | 4.26 | 10.84 | 203 | 23.4 | 11.02 |
| 23 | protein-L-isoaspartate O-methyltransferase isoform 8 | 2.88 | 10.37 | 164 | 17.7 | 6.54 |
| 24 | glutathione S-transferase Mu 4 isoform X2 | 2.53 | 5.96 | 151 | 17.4 | 6.52 |
| 25 | 60S ribosomal protein L35 | 2.49 | 8.13 | 123 | 14.5 | 11.05 |
| 26 | ras-related protein Rab-11A | 2.17 | 5.09 | 216 | 24.4 | 6.57 |
| 27 | ras-related protein Rab-14 | 2.13 | 11.16 | 215 | 23.9 | 6.21 |
| 28 | high mobility group protein B2 | 2.03 | 9.05 | 210 | 24.1 | 7.31 |
| 29 | synaptogyrin-2 | 1.94 | 4.46 | 224 | 24.8 | 4.78 |
| 30 | four and a half LIM domains protein 2 | 1.93 | 3.23 | 279 | 32.1 | 7.3 |
| 31 | heat shock cognate 71 kDa protein isoform 2 | 1.8 | 1.28 | 627 | 68.7 | 5.52 |
| 32 | ubiquitin-conjugating enzyme E2 K isoform 3 | 1.78 | 7.69 | 130 | 14.4 | 6.54 |
| 33 | rho GDP-dissociation inhibitor 1 | 1.78 | 7.84 | 204 | 23.4 | 5.2 |
| 34 | polypyrimidine tract-binding protein 1 isoform 3 | 1.73 | 1.84 | 489 | 52.6 | 9.03 |
| 35 | 60S ribosomal protein L15 | 1.66 | 3.43 | 204 | 24.1 | 11.62 |
| 36 | polyubiquitin-C | 1.64 | 8.17 | 734 | 82.5 | 8.62 |
| 37 | 3-hydroxyacyl-CoA dehydrogenase type-2 | 0 | 3.83 | 261 | 27.3 | 8.76 |
| 38 | RNA transcription, translation and transport factor protein | 0 | 3.69 | 244 | 28.1 | 6.89 |
| 39 | 60S ribosomal protein L13 | 0 | 3.79 | 211 | 24.3 | 11.55 |
